# TMAO-Activated Hepatocyte-Derived Exosomes Are Widely Distributed in Mice with Different Patterns and Promote Vascular Inflammation

**DOI:** 10.1155/2022/5166302

**Published:** 2022-02-14

**Authors:** Xiang Liu, Jiazichao Tu, Ziqin Zhou, Bingxin Huang, Jianrong Zhou, Jimei Chen

**Affiliations:** ^1^Department of Cardiac Surgery, Guangdong Cardiovascular Institute, Guangdong Provincial People's Hospital, Guangdong Academy of Medical Sciences, Guangzhou 510080, China; ^2^Guangdong Provincial Key Laboratory of South China Structural Heart Disease, Guangzhou 510080, China

## Abstract

**Background:**

Trimethylamine-N-oxide (TMAO) has been shown to be an important player in cardiovascular disease (CVD) by promoting vascular inflammation and endothelial dysfunction. We recently found that exosomes (Exos) released from TMAO-activated hepatocytes (TMAO-Exos) could significantly induce inflammation and endothelial dysfunction. However, understandings of how are the Exos secreted by hepatocytes, where are they distributed *in vivo*, and what effects will they have on vascular inflammation remain limited. The present study aimed to explore the hub genes involved in the production of TMAO-Exos and their distributions *in vivo* and effects on inflammation.

**Methods:**

The transcriptome profiles of primary rat hepatocytes stimulated with TMAO were obtained from the GSE135856 dataset in the Gene Expression Omnibus repository, and the hub genes associated with Exos were screened and verified by qPCR. Next, Exos derived from TMAO-treated hepatocytes were isolated using differential centrifugation and given intravenously to mice. After 24 h, the distributions of DiI-labelled Exos were visualized with a fluorescence microscope, and the levels of proinflammatory genes in the aorta were detected by qPCR.

**Results:**

*Phgdh*, *Mdh*2, *Echs*1, *Rap*2*a*, *Gpd*1*l*, and *Slc3a*2 were identified as hub genes that may be involved in the production of TMAO-Exos. And TMAO-Exos were found to be efficiently taken up by cardiomyocytes, hepatocytes, and endothelial cells in the aorta and gastrocnemius muscle. Furthermore, TMAO-Exos, but not control-Exos, could significantly promote the mRNA expressions of *Tnf*, *Icam*1, *Sele*, and *Cox-*2 in the aorta.

**Conclusions:**

We provided clues about how TMAO may stimulate hepatocytes to produce Exos and further offered evidence that Exos secreted by TMAO-treated hepatocytes could be widely distributed *in vivo* and promote vascular inflammation.

## 1. Introduction

There is increasing evidence that gut microbiota and its metabolites play a key role in the pathogenesis and development of cardiovascular disease (CVD) [1]. Thereinto, trimethylamine-N-oxide (TMAO) has been found to be an independent risk factor for adverse cardiovascular events [2–6], which may be related to excessive vascular inflammation and endothelial dysfunction provoked [7–10]. It has been shown that dietary precursors such as choline, betaine, and L-carnitine can be metabolized into trimethylamine in the gut flora and further catalyzed into trimethylamine-N-oxide (TMAO) in the liver [2, 5, 11]. However, the mechanisms underlying TMAO are still not completely understood.

Exosomes (Exos) have gained a growing concern for the pivotal roles in cardiovascular physiology [12]. Exos are nanosized membrane vesicles produced by nearly all types of cells, ranging from approximately 30 to 100 nm in diameter and containing a variety of bioactive molecules [12–14]. Recent research has found that Exos derived from hepatocytes play an important role in inflammation, endothelial function, and metabolic disorders [15–19]. In the latest work, we found that Exos secreted by TMAO-treated hepatocytes (TMAO-Exos) contained a distinctive profile of miRNAs compared to those from the TMAO-free group (control-Exos), and furthermore, TMAO-Exos could notably promote inflammation, damage vascular endothelial cells (VECs), and impair endothelium-dependent vasodilation [20]. However, understandings of how these Exos are produced by hepatocytes, where are they distributed *in vivo*, and what effects will they have on vascular inflammation remain limited.

In the present study, we first obtained the transcriptome profiles of primary rat hepatocytes stimulated with or without TMAO [21] and then identified the hub genes related to Exos. Next, Exos were isolated from hepatocytes treated with or without TMAO and given intravenously to mice, and then their distributions in the heart, liver, aorta, and gastrocnemius muscle and effects on vascular inflammation were examined.

## 2. Materials and Methods

### 2.1. Hepatic Gene Expressions Induced by TMAO

The transcriptome profiles of primary rat hepatocytes stimulated with or without TMAO (50 *μ*mol/L) for 30 h were obtained from the GSE135856 dataset in the Gene Expression Omnibus repository [21, 22]. The microarray experiments were performed using a single Affymetrix Clariom S Rat array (Affymetrix, Santa Clara, CA, USA) with three biological repetitions. The open-source Bioconductor packages, affy and limma, were used to process the data [21].

### 2.2. Enrichment Analysis

The differentially expressed genes (*P* < 0.05) related to Exos were screened and visualized on a heatmap constructed in R. DAVID (Database for Annotation, Visualization, and Integrated Discovery) was used for investigating the functional annotation of genes. GO (gene ontology) analysis was performed to elaborate the biological process, STRING (Search Tool for the Retrieval of Interacting Genes/Proteins) database (v11.0) [23] was used for analyzing the protein-protein interaction (PPI), and networks were constructed on Cytoscape platform (v3.8.2) [24]. CytoHubba plugin was used to identify hub genes with a threshold value >0.4, and the color of the nodes represented the degree of gene interaction.

### 2.3. Cell Culture and Exos Isolation

The procedures were performed as described in our recent study [20]. In brief, AML12 cells (iCell Bioscience Inc., Shanghai) were cultured in DMEM/F12 (iCell Bioscience Inc., Shanghai) containing Exos-depleted serum (ViVaCell, Shanghai) and treated with TMAO (Tokyo Chemical Industry Co., Ltd.) at a physiological concentration of 50 *μ*mol/L for 48 h (TMAO-Exos). The untreated group served as the control (control-Exos). Exos were isolated and purified from the culture supernatant using differential centrifugation and then resuspended in PBS.

### 2.4. Exos Identification and Labelling

The protein levels of the Exos were measured using the BCA protein assay kit (Thermo Fisher Scientific, MA, USA). The ultrastructure of the Exos was inspected using a transmission electron microscope (JEM 1200-EX, Japan). In brief, Exos suspensions were loaded on 200-mesh formvar-coated grids and then negatively stained with phosphotungstic acid. The samples were observed under a transmission electron microscope at a voltage of 100 kV. The concentration and size distribution of the Exos were detected by nanoparticle tracking analysis (Nanosight NS300, Malvern, UK). Exosomal markers of CD9 and TSG101 and the negative marker of calnexin were detected by western blotting. In brief, the samples were separated by SDS-PAGE and transferred onto Millipore polyvinylidene difluoride membranes. The membranes were incubated overnight at 4°C with the primary antibodies of CD9 (Zenbio, Chengdu, China), TSG101 (Zenbio, Chengdu, China), and calnexin (Affinity Biosciences, Jiangsu, China) and visualized with enhanced chemiluminescence reagent (Millipore, MA, USA). Exos were labelled with DiI (Beyotime Biotechnology, Shanghai, China) for *in vivo* tracer experiments.

### 2.5. Animal Experiments

All experiments conform to the protocols approved by the Institutional Animal Care and Use Committee of Guangdong Provincial People's Hospital. Male wild-type C57BL/6 mice (six weeks old) were purchased from the Experimental Animal Center of Sun Yat-sen University. The mice were intravenously injected with 30 *μ*g of Exos resuspended in 100 *μ*L of PBS. After 24 h, the mice were anesthetized by pentobarbital sodium (50 mg/kg), and then the hearts, thoracic aortas, livers, and gastrocnemius muscles were collected for subsequent study.

### 2.6. Fluorescence Detection

The hearts, thoracic aortas, livers, and gastrocnemius muscles were fixed in 4% paraformaldehyde and then dehydrated and embedded in paraffin. Samples underwent dewaxing and antigen retrieval. The slides were blocked in 10% goat serum for 30 min at room temperature and then incubated with the primary antibody of CD31 (Abcam, MA, USA) overnight. Slides were incubated with Alexa Fluor^®^ 488 donkey anti-rabbit IgG (H + L). Wheat germ agglutinin (WGA, Sigma-Aldrich, USA) staining was used to outline the cardiomyocytes. Slides were then washed and stained with DAPI (Solarbio, Beijing, China). The positive signals were detected with a fluorescence microscope (Olympus, Tokyo, Japan).

### 2.7. Quantitative Polymerase Chain Reaction

Quantitative polymerase chain reaction (qPCR) was performed as described in our previous study [25]. In brief, total RNA was extracted using TRIzol reagent (Invitrogen, USA), and concentration was measured using the NanoDrop 2000 spectrophotometer (Thermo Fisher Scientific, MA, USA). Then, RNA was reversely transcribed into cDNA using Color Reverse Transcription Kit (EZBioscience, CA, USA), and qPCR was performed on Bio-Rad CFX-96 (Bio-Rad, CA, USA) with Color SYBR Green qPCR Master Mix (EZBioscience, CA, USA). The expressions were normalized to Gapdh. The qPCR primers used in the study are listed in Table 1.

### 2.8. Statistical Analysis

Statistical analysis was conducted using SPSS 20.0 software (SPSS Inc., Chicago, IL, USA), and the graphs were plotted by GraphPad Prism (version 9.3.0, San Diego, USA). Data were presented as mean ± standard error of the mean (SEM). For continuous variables with normal distribution, the comparisons between two groups were performed with independent *t*-test. For continuous variables with nonnormal distribution, the comparisons between two groups were performed with Wilcoxon rank-sum test. A *P* value <0.05 was considered statistically significant.

## 3. Results

### 3.1. Exos-Related Genes in Hepatocytes Were Dysregulated by TMAO

**Figure 1:**
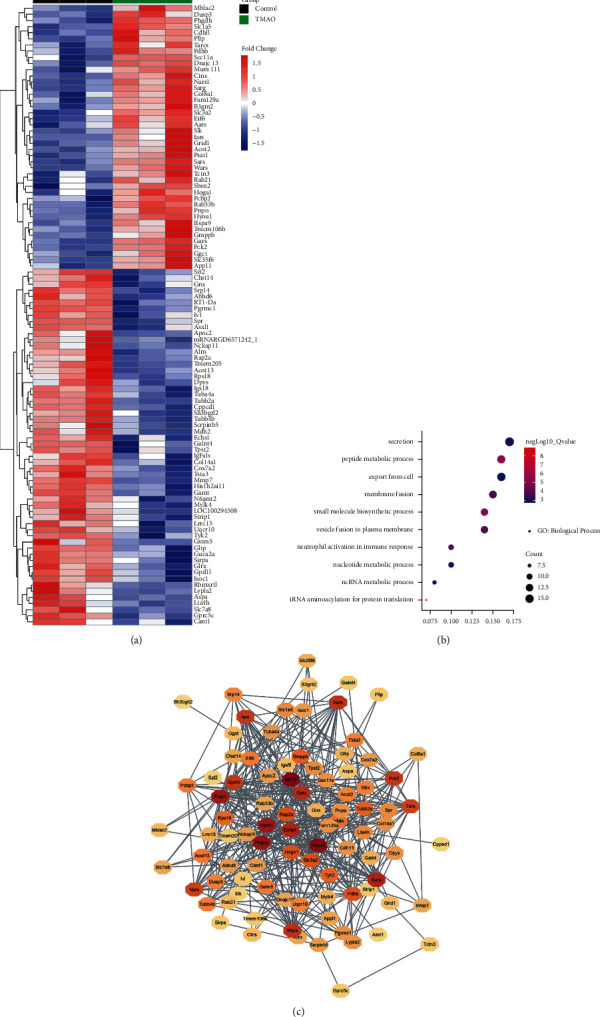
Exos-related genes in hepatocytes were dysregulated by TMAO. (a) A total of 101 genes related to Exos changed significantly (*P* < 0.05) after TMAO treatment. (b) The differentially expressed genes were significantly enriched in the biological processes of secretion, peptide metabolic process, export from the cell, membrane fusion, and small-molecule biosynthetic process. (c) *Phgdh*, *Mdh*2, *Echs*1, *Rap*2*a*, *Gpd*1*l*, *Slc*3*a*2, *Hyou*1, *Hspa*9, *Psat*1, and *Pck*2 were identified as the top 10 hub genes.

Microarray assay was performed to determine the expression profiles of genes in primary rat hepatocytes stimulated with TMAO. Compared to the untreated group, a total of 101 genes related to Exos changed significantly (*P* < 0.05), and among these genes, 43 were upregulated and 58 were downregulated (Figure 1(a)). GO analysis was used to explore the predominant biological processes, and the results showed that the differentially expressed genes (DEGs) were significantly enriched in secretion, peptide metabolic process, export from the cell, membrane fusion, and small-molecule biosynthetic process (Figure 1(b)). Besides, the PPI networks were constructed to further elaborate the interactions among the DEGs and identified *Phgdh*, *Mdh*2, *Echs*1, *Rap*2*a*, *Gpd*1*l*, *Slc*3*a*2, *Hyou*1, *Hspa*9, *Psat*1, and *Pck*2 as the top 10 hub genes (Figure 1(c)).

### 3.2. Verification of the Hub Genes

**Figure 2:**
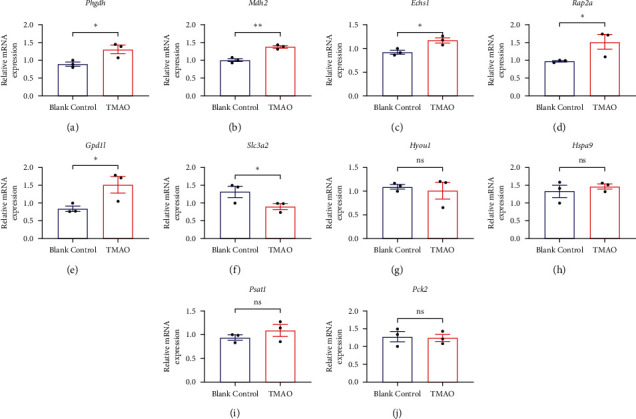
Verification of the top 10 hub genes. (a–e) TMAO remarkably enhanced the mRNA expressions of *Phgdh*, *Mdh*2, *Echs*1, *Rap*2*a*, and *Gpd*1*l* and (f) reduced *Slc*3*a*2 levels. (g–j) *Hyou*1, *Hspa*9, *Psat*1, and *Pck*2 remained unchanged. qPCR was used to determine the mRNA expressions (*n* = 3). ^*∗*^*P* < 0.05 vs. the blank control group. ns indicates not significant.

To verify the changes of the hub genes, AML12 cells were stimulated with or without TMAO for 48 h, and the mRNA levels of the top 10 hub genes were detected by qPCR. It was shown that TMAO remarkably enhanced the mRNA expressions of *Phgdh*, *Mdh*2, *Echs*1, *Rap*2*a*, and *Gpd*1*l* (Figures 2(a)–2(e)) and reduced *Slc*3*a*2 levels (Figure 2(f)). However, *Hyou*1, *Hspa*9, *Psat*1, and *Pck*2 remained unchanged (Figures 2(g)–2(j)).

### 3.3. Isolation and Identification of Exos

**Figure 3:**
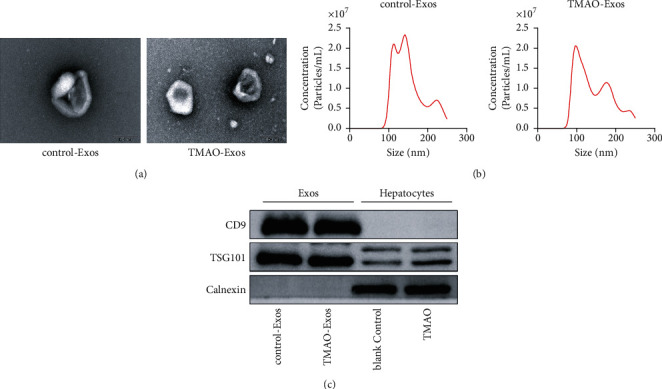
Isolation and identification of Exos. (a) Nanovesicles with a cup-shaped morphology and a typical size around 100 nm were isolated and purified from the cell culture supernatant. Bar: 100 nm. (b) The size distribution of the Exos showed no significant difference between control-Exos and TMAO-Exos. (c) Exosomal markers of CD9 and TSG101 were enriched in Exos groups, and the negative marker of calnexin was detected only in the whole cell lysate.

Nanovesicles with a cup-shaped morphology and a typical size around 100 nm were isolated and purified from the cell culture supernatant (Figure 3(a)). The size distribution profiles and concentrations of the Exos showed no significant differences between the two groups (Figure 3(b)). Exosomal markers of CD9 and TSG101 were mainly enriched in control-Exos and TMAO-Exos, and the negative marker of calnexin was detected only in the whole cell lysate (Figure 3(c)).

### 3.4. Exos Were Widely Distributed *In Vivo* with Different Patterns

**Figure 4:**
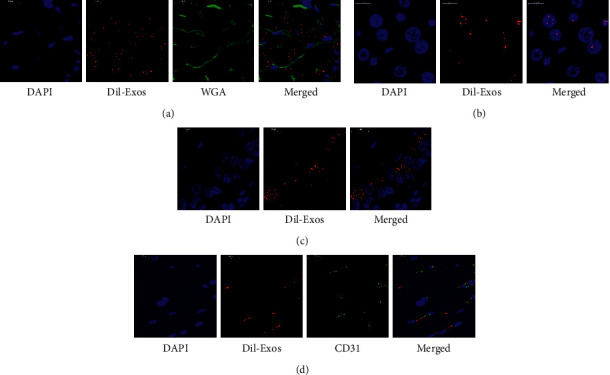
Exos were widely distributed *in vivo* with different patterns. Exos secreted by TMAO-treated hepatocytes could be efficiently captured by (a) cardiomyocytes, (b) hepatocytes, and (c) endothelial cells in the aorta. (d) The Exos appeared to keep themselves out of the skeletal muscle cells and preferentially located in the endothelial cells.

TMAO-Exos were labelled with DiI and administered intravenously. The results clearly showed that Exos could be efficiently taken up by cardiomyocytes (Figure 4(a)), hepatocytes (Figure 4(b)), and endothelial cells in the aorta (Figure 4(c)). It was worth noting that these Exos were predominantly localized in the nuclei of hepatocytes. And unlike in cardiomyocytes, these Exos appeared to keep themselves out of the skeletal muscle cells and preferentially located in the endothelial cells, as indicated by the same subcellular localizations of Exos and CD31 proteins in the gastrocnemius muscle (Figure 4(d)).

### 3.5. TMAO-Exos Promoted Vascular Inflammation

**Figure 5:**
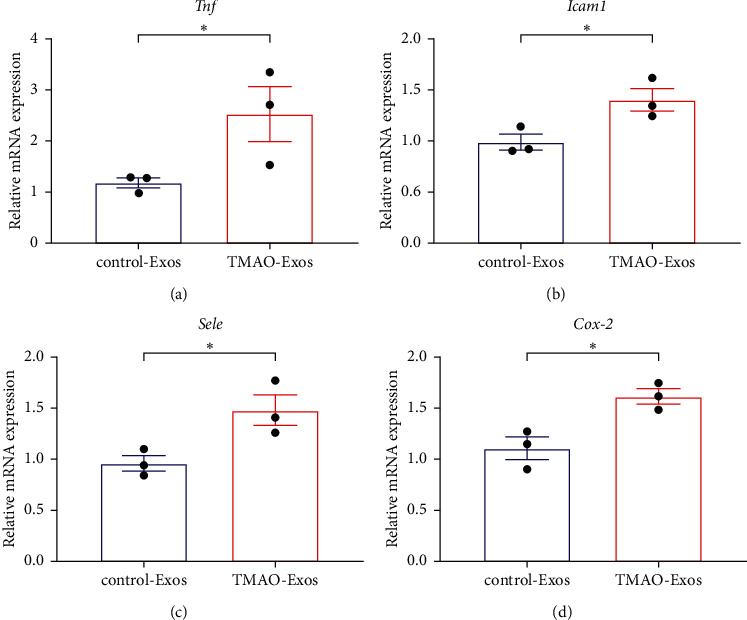
TMAO-Exos promoted the mRNA expressions of inflammatory genes in aortas. TMAO-Exos notably enhanced the mRNA levels of (a) *Tnf*, (b) *Icam*1, (c) *Sele*, and (d) *Cox-*2. qPCR was used to determine the mRNA expressions (*n* = 3). ^*∗*^*P* < 0.05 vs. the control-Exos group.

The *in vivo* tracer experiments clearly showed that TMAO-Exos could target endothelial cells in the aorta and gastrocnemius muscle, so we assessed the effects of TMAO-Exos on the mRNA expressions of inflammatory genes in aortas. Compared to control-Exos, TMAO-Exos significantly promoted the mRNA expressions of *Tnf*, *Icam*1, *Sele*, and *Cox-*2 (Figures 5(a)–5(d)).

## 4. Discussion

In the current work, we first identified the hub genes in hepatocytes after TMAO treatment, which may be related to the production of TMAO-Exos. Next, we provided evidence that TMAO-Exos could be taken up by several tissues including the heart, liver, aorta, and gastrocnemius muscle with different distribution patterns. And finally, we found that TMAO-Exos, but not control-Exos, could significantly promote the mRNA expressions of *Tnf*, *Icam*1, *Sele*, and *Cox-*2 in the aorta.

There is a close relationship between TMAO and the liver. TMAO, at a physiological concentration of 50 *μ*mol/L, has been shown to be able to target hepatocytes and result in the abnormal expressions of hepatic genes, thus exerting an influence on metabolic disorder [21]. Moreover, we recently found that TMAO (50 *μ*mol/L) could stimulate hepatocytes to release functional Exos [20]. However, the molecular pathways and hub genes behind it remain unknown. It was found that the fusion of multivesicular bodies (MVBs) with the plasma membrane is critical for the extracellular secretion of Exos, and intracellular molecules and pathways such as Rab27a/b and V-ATPase-mediated acidification of MVBs are implicated [26]. An interference study showed that HGS, Alix, TSG101, and nSmase2 were necessary for the Exos production by normal or ethanol-treated hepatocytes [27]. And other studies revealed that Tsg101 and Vps4a played a pivotal role in exosomal secretion [28, 29]. Based on the aforementioned study [21], a Exos-related gene set was selected to identify the predominant biological processes and hub genes which responded to TMAO treatment. The results showed that the DEGs were mainly enriched in secretion, peptide metabolic process, export from the cell, membrane fusion, and small-molecule biosynthetic process, which suggested that these DEGs may be related to the synthesis and secretion of TMAO-Exos. Furthermore, we verified that *Phgdh*, *Mdh*2, *Echs*1, *Rap*2*a*, and *Gpd*1*l* were upregulated, and *Slc*3*a*2 was downregulated by TMAO. *Phgdh* and *Mdh*2 are classified into the biological process of “small-molecule biosynthetic process.” The protein encoded by *Phgdh* is essential for serine synthesis and contributes to glucose and lipid homeostasis [30–32]. And gene *Mdh*2 encodes a mitochondrial enzyme that plays a pivotal role in the malate-aspartate shuttle and glucose homeostasis [33, 34]. In addition, *Slc*3*a*2 encodes a membrane protein and is involved in amino acid transport and endoplasmic reticulum stress [35–37]. However, the results simply provided some early clues about how TMAO may stimulate hepatocytes to produce Exos, and the exact mechanisms remain to be further elucidated by using the gain- and loss-of-function strategies.

Similarly, the distribution patterns of Exos remain poorly defined. It was suggested that the processes may depend on the distinctive factors or receptors located on the recipient cell surfaces [38]. However, Horibe et al. found that Exos could be nonselectively incorporated into recipient cells via a different mechanism, which may depend on the recipient cells rather than the donor cells [39]. In the present work, we showed that TMAO-Exos could be distributed in tissues including the heart, liver, aorta, and gastrocnemius muscle after systemic administration, suggesting that these Exos spread widely *in vivo* through the bloodstream. However, it seemed that the distribution of these Exos varied depending on the recipient cell type, as indicated by the observations that they could be incorporated by cardiomyocytes, but appeared to keep themselves out of the skeletal muscle cells and preferentially located in the endothelial cells. In addition, it was interesting to see that these Exos were able to be captured by hepatocytes and localized in the nuclei. These findings were consistent with previous research, in which Exos were able to be captured by the donor cells themselves [27, 39]. Furthermore, we found that TMAO-Exos could significantly promote the mRNA expressions of *Tnf*, *Icam*1, *Sele*, and *Cox-*2 in the aortas. These proinflammatory genes have been shown to play pivotal roles in the regulation of inflammation, endothelial dysfunction, and atherosclerosis [40–43].

## 5. Conclusions

In the current study, we provided clues about how TMAO may stimulate hepatocytes to produce Exos and further offered evidence that Exos secreted by TMAO-treated hepatocytes could be widely distributed *in vivo* and promote vascular inflammation. These findings may help to advance our understanding of the mechanisms by which TMAO promotes CVD.

## Figures and Tables

**Table 1:** qPCR primers for mRNA (*Mus musculus*) used in the study.

Name	Forward sequence	Reverse sequence
Phgdh	CCAGGTGGTTACACAAGGAACA	TTCACGTCTGCCTGCTTAGATG
Mdh2	TTTGTGGCAGAGCTAAAGGGTT	GTACACTGAGAGATCAGGGGGA
Echs1	AATGGAGATGGTCCTCACTGGT	TCTGCACATTGGATGGCTTCTT
Rap2a	CAGCAGAGCTTCCAAGACATCA	CTCTCCAGGTCCACTTTGTTCC
Gpd1l	TCCGACATCATCCGAGAGAAGA	AAGGCCGTTCTGCATCACTTT
Slc3a2	GGCCCAATTCACAAGAACCAGA	TGGGAGTGAGGTCCAAAATGATG
Hyou1	GATCTTCGGGTATTTGGCTCCC	TAAAGTGGGCCTTGATGCCTTT
Hspa9	AACTCCTGTGTGGCTGTTATGG	CAAGTCGTTCTCCATCTGCTGT
Psat1	GGGTGGAGTTTGACTTCGTACC	TCTTCTGAGCACCAGCGAAAAT
Pck2	GAGGCTGAGAACACTGCCATAC	TGCGAAGGAGTTACAATCACCG
Tnf	CTGTAGCCCACGTCGTAGC	TTGAGATCCATGCCGTTG
Icam1	CCCACGCTACCTCTGCTC	GATGGATACCTGAGCATCACC
Sele	ATGCCTCGCGCTTTCTCTC	GTAGTCCCGCTGACAGTATGC
Cox-2	TTCAACACACTCTATCACTGGC	AGAAGCGTTTGCGGTACTCAT
Gapdh	ACTCTTCCACCTTCGATGCC	TGGGATAGGGCCTCTCTTGC

Phgdh: 3-phosphoglycerate dehydrogenase; Mdh2: malate dehydrogenase 2, NAD (mitochondrial); Echs1: enoyl coenzyme A hydratase, short chain, 1, mitochondrial; Rap2a: Ras-related protein 2a; Gpd1l: glycerol-3-phosphate dehydrogenase 1-like; Slc3a2: solute carrier family 3 (activators of dibasic and neutral amino acid transport), member 2; Hyou1: hypoxia upregulated 1; Hspa9: heat shock protein 9; Psat1: phosphoserine aminotransferase 1; Pck2: phosphoenolpyruvate carboxykinase 2 (mitochondrial); Tnf: tumor necrosis factor; Icam1: intercellular adhesion molecule-1; Sele: selectin, endothelial cell; Cox-2: cyclooxygenase-2; Gapdh glyceraldehyde-3-phosphate dehydrogenase.

## Data Availability

The data relevant to this study are available from the corresponding author upon reasonable request.
